# The relationship between consumption of tyrosine and phenylalanine as precursors of catecholamine at breakfast and the circadian typology and mental health in Japanese infants aged 2 to 5 years

**DOI:** 10.1186/1880-6805-32-13

**Published:** 2013-10-01

**Authors:** Osami Akimitsu, Kai Wada, Teruki Noji, Nozomi Taniwaki, Milada Krejci, Miyo Nakade, Hitomi Takeuchi, Tetsuo Harada

**Affiliations:** 1 Laboratory of Environmental Physiology, Graduate School of Integrated Arts and Sciences, Kochi University, Kochi, Japan; 2 Department of Physical Education, Faculty of Education, Kochi University, Kochi, Japan; 3 Affiliated Kindergarten, Faculty of Education, Kochi University, Kochi, Japan; 4 Department of Health Education, Faculty of Education, University of South Bohemia, Ceske Budejovice, Czech Republic; 5 Department of Nutritional Management, Faculty of Health and Nutrition, Tokai Gakuen University, Nagoya, Japan

**Keywords:** Birth season, Circadian typology, Infants aged 2 to 5 years

## Abstract

**Background:**

This study aims to examine the relationship between tyrosine and phenylalanine intake at breakfast as precursors of dopamine, and scores on the Torsvall-Åkerstedt Diurnal Type Scale and of mental health in Japanese infants aged 2 to 5 years.

**Results:**

An integrated questionnaire was administered to parents of 1,367 infants attending one of ten nursery schools governed by Kochi City or a kindergarten affiliated with the Faculty of Education at Kochi University (775 answers for analysis: 56.7%) in May and June 2008. Questionnaires included the Torsvall-Åkerstedt Diurnal Type Scale and questions on sleep habits (onset, offset, quality, quantity, and so on), meal habits (content and regularity of timing), and mental health (depressive states). Amount of tyrosine and phenylalanine intake was calculated based on a breakfast content questionnaire and data on the components of amino acids in foods. Infants who ingested more than 800 mg of tyrosine or phenylalanine at breakfast per meal were more morning-type than those who ingested less than 800 mg (ANOVA: *P*= 0.005). However, this relationship disappeared in the ANCOVA analysis (with the covariance of tryptophan intake, *P*= 0.894). Infants who ingested more than 800 mg of the two amino acids at breakfast showed significantly higher mental health scores (lower frequency of depressive states) than those who ingested less than 800 mg (ANOVA: *P* = 0.004). This relationship remained significant when ANCOVA analysis was performed with the covariance of tryptophan (ANCOVA: *P*= 0.017).

**Conclusions:**

These results suggest that tyrosine and phenylalanine ingested at breakfast are not related with circadian phase, but are relate with mental health in infants.

## Introduction

Harada [1] argued that, based on various epidemiological data, diurnal rhythms and sleep health in infants aged 2 to 6 years may be highly sensitive to the living environment, including light, regularity, and timing of meals in comparison with university students as adults. Breakfast content is also strongly linked to sleep health. An example of this can be found in tryptophan, an essential amino acid that is absorbed exclusively from meals. It is metabolized to melatonin via 5-hydroxytryptamine (serotonin) by a series of four enzymes in the pineal body, in which serotonin synthesis can be supported by Vitamin B6 as a cofactor in the tryptophan-serotonin metabolic pathway [2,3]. Tryptophan intake at breakfast has been shown to promote morning-type circadian typology and higher sleep quality in Japanese babies and infants aged 0 to 6 [4]. In the metabolism line of tryptophan-serotonin-melatonin, Waldhauser *et al*. [5] reported that concentration of plasma melatonin shown by infants aged 1 to 7 years were 500 to 800 pg/ml and five to eight times higher than that of adults (about 100 pg/ml). Serotonin levels in the brain of infants could be hypothesized to be much higher than that in adults.

**Figure 1:**
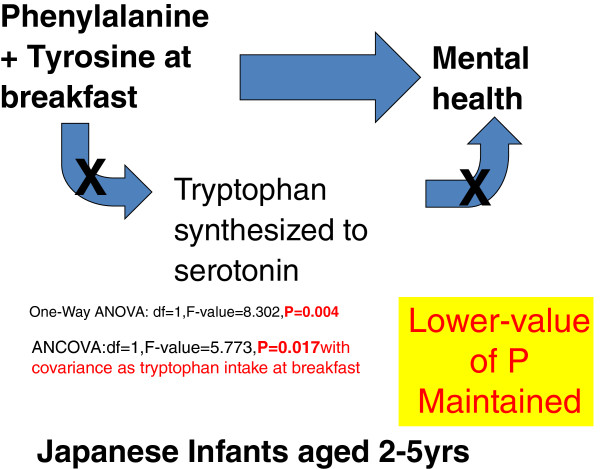
Mental health may be related to phenylalanine and tyrosine intake at breakfast via metabolism to dopamine in the human brain.

Catecholamine level has been reported to relate to mood, depression, and immune parameters. For example, catecholamine depletion induced by the administration of α-methylparatyrosine (AMPT) triggered a transient exacerbation of depressive symptoms as well as a transient reduction of plasma sIL-4 level in patients with seasonal affective disorder who were in remission after using light therapy [6]. Another study showed that catecholamine depletion due to AMPT application resulted in decreased happiness, euphoria, energy, talkativeness, vigor, and attentiveness, and in increased sleepiness, fatigue, sedation, and eye blink rate in healthy adults [7]. Irwin *et al*. [8] reported that insomniacs showed increased nocturnal norepinephrine and decreased nocturnal natural killer cell responses. Measurement of norepinephrine concentration included in cerebrospinal fluid in monkeys showed circadian variation of catecholamine metabolism, with high metabolism in light 12 hours and low in dark 12 hours [9]. Unlike 5HTP, DOPA synthesis rates in the hypothalamus, hippocampus, and cortex showed no significant variation with meal conditions in rats [10]. In human beings, it has not yet been studied whether intake of tyrosine and phenylalanine from meals affects mental health, sleep health, or circadian typology, especially in infants. One working hypothesis, that intake of tyrosine and phenylalanine from breakfast taken by infants relates with their mental health could be proposed (Figure 1). This study aimed to test this working hypothesis.

### Participants and methods

The child version [4] of the Torsvall-Åkerstedt Diurnal type scale (Table 1) [11] was administered to 1,367 parents of infants (all born and grown in Kochi City where 0.34 million people are living). Answers were obtained for 845 infants (435 female, 410 male) aged between 2 and 5 years, who were attending a nursery school or kindergarten in 2008 (giving a 61% response rate). Of these, 70 answers were excluded from the statistic analysis because of incomplete responses, leaving 775 answers for analysis, with a response rate of 56.7%. Parents completed the questionnaires for infants aged 2–5 years. More than 95% of children aged 2 to 5 years were born and grew up in Kochi City. All kindergartens were located in Kochi City, Japan (33°N).

**Table 1:** **A version modified for infants**[4]**of the Torsvall-Åkerstedt Diurnal type scale**[11]

**Questions**	**Answers on a four-point scale**			
	**4**	**3**	**2**	**1**
1. When would your child prefer to wake up (provided your child has a full day’s play of 8 h) if your child were totally free to arrange his (her) time?	Before 6:30 a.m.	6:30 to 7:29 a.m.	7:30 to 8:29 a.m.	8:30 a.m. or later
2. When would your child prefer to go to bed (provided your child has a full day’s play of 8 h) if your child were totally free to arrange his (her) time?	Before 9:00 p.m.	9:00 to 9:59 p.m.	10:00 to 10:59 p.m.	11:00 p.m.
3. If your child always had to go to bed at 9:00 p.m., how easy would it be to fall asleep?	Easy: would fall asleep almost instantly	Somewhat easy: would lie awake for a short time	Somewhat difficult: would lie awake for some time	Very difficult: would lie awake for a long time
4. If your child always had to wake up at 6:00 a.m., how easy would it be to wake up?	Easy: no problem at all	A little unpleasant but no big problem	Somewhat difficult and unpleasant	Very difficult and unpleasant
5. When does your child usually begin to feel the first signs of tiredness and need for sleep?	Before 9:00 p.m.	9:00 to 9:59 p.m.	10:00 to 10:59 p.m.	11:00 p.m. or later
6. How long does it usually take before your child feels fully awake in the morning after waking up?	1 to 10 min	11 to 20 min	21 to 40 min	more than 40 min
7. Please indicate to what extent your child is a morning or evening person.	Pronounced morning person (morning alert and evening tired)	Somewhat morning person	Somewhat evening person (morning tired and evening alert)	Pronounced evening person

#### *Questionnaire content*

In this study, the Torsvall-Åkerstedt Diurnal Type Scale, which consists of seven questions, was used to estimate circadian typology instead of the morningness-eveningness questionnaire of Horne and Östberg [12], which consists of 19 questions, because the Japanese kindergarten arrangement meant that there was only a limited time for the participants to answer questions. High positive correlations (more than 0.8) have been recorded between the scores of the two questionnaires, which had been administered to the same population of university students (Harada *et al*., unpublished work) and children (Ishihara, personal communication). The version developed for children [4] from the original version of the scale [11] was used to measure diurnal preference subjectively. The original Torsvall-Åkerstedt questionnaire [11] consisted of seven questions: three pertaining to sleep onset, three to sleep offset, and one to peak timing of activity. Each question allows for choice (scored from 1 to 4) and the score of the Torsvall-Åkerstedt Diurnal Type Scale was given as the sum of the seven answers. Scores ranged from 7 to 28, with lower scores representing evening-types and higher scores representing morning-types. The Japanese version for infants aged 2 to 5 years has been used in several papers [4,13-15] and was again used in this study.

Questions on breakfast habits included regularity of breakfast timing, frequency of having a breakfast that consisted of a staple food (carbohydrate), a main dish (protein), and a side dish (vitamins and minerals) (Table 2a), and the contents of foods regularly eaten for breakfast (Table 2b).

**Table 2:** **Questions about breakfast content (a) and question on frequency of taking nutritionally well-balanced breakfast (b) and answers after Gomyo and Hasegawa**[17]

**(a): Q. Indicate all the types of foods that your child regularly eats for breakfast. For numbers 11, 13, 14, 22, 29, please write the type of food often eaten (for example, beef, mackerel, herring, banana)**	**(b): Q. How frequently does your child take a nutritionally well-balanced breakfast that consists of carbohydrates, a main dish (protein resources) and a side dish (vitamin and minerals)?**
(1)Rice	**A.**
(2)Bread	(1) Everyday,
(3)Noodles	(2) 4 or 5 days/week,
(4)Potatoes	(3) 2 or 3 days/week,
(5)Cereal	(1) 0 or 1 day/week
(6) Eggs	
(7) Fermented soybeans ('natto’)	
(8) Tofu	
(9) Soymilk	
(10) Miso soup	
(11) Meat (____________)	
(12) Processed meat (for example, ham, bacon)	
(13) Fish (____________)	
(14) Dried fish (____________)	
(15) Seaweed	
(16) Milk	
(17) Milk products (for example, yogurt, cheese)	
(18) Lactic acid drink	
(19) Brightly colored vegetables	
(20) Other vegetables	
(21) 100% vegetable juice	
(22) Fruit (___________)	
(23) 100% fruit juice	
(24) 100% vegetable and fruit juice	
(25) Coffee	
(26) Black tea	
(27) Green tea	
(28) Other juice	
(29) Nutritional supplements (_________)	

#### *Method for calculation of the estimated tyrosine and phenylalanine intake*

This method was the same as the authorized method for calculation of the estimated tryptophan intake [16]. The calculation was made only for the children who took breakfast every day or nearly every day, no matter what the contents. Those infants who often skipped breakfast were deleted from the list for the calculation.

The amount of tyrosine or phenylalanine contained in 100 g of food was estimated based on tables of the contents of amino acids in all kinds of foods by Gomyo and Hasegawa [17] (Table 3). The amount of foods taken by infants were estimated from the recommended amount of breakfast foods for infants aged 2 to 5 years and general breakfast menu most frequently taken by Japanese infants [18,19]. The amount of tyrosine or phenylalanine taken in one breakfast was calculated from these two estimations (**a**-value).

**Table 3:** Suggested food items divided by food category and composition values (per 100 g)

**Category**	**Dishes**	**Foods**	**Weight (g)**	**Energy (kcal)**	**Protein (g)**	**Tryptophan (mg)**	**Vitamin B6 (mg)**	**Phenylalanine (mg)**	**Tyrosine PI (mg)**
Carbohydrate		Rice	100	168	2.5	36	0.02	138	105
		Bread	100	264	9.3	96	0.03	461	238
		Noodles	100	105	2.6	28	0.01	141	77
		Cereal	100	381	7.8	41	0.04	411	287
Main dish	Meat	Meat	100	142	21.6	259	0.41	884	754
		Processed meat	100	321	13.2	143	0.10	506	401
	Fish	Fish	100	158	28.9	327	0.37	1137	976
		Dried fish	100	187	30.1	262	0.15	868	749
	Eggs	Eggs	100	151	12.3	184	0.08	629	511
	Soybeans	Fermented beans	100	200	16.5	242	0.24	866	693
		Tofu	100	72	6.6	100	0.05	369	277
		Soy milk	100	64	3.2	47	0.05	173	134
Side dish	Vegetable	Brightly colored vegetables	100	29	1.4	20	0.13	51	33
		Other vegetable	100	23	1.0	8	0.10	24	15
	Roots	Potatoes	100	104	1.4	17	0.23	66	35
	Seaweed	Seaweed	100	188	41.4	510	0.59	1523	1324
	Soup	Soybean soup	100	192	12.5	140	0.11	678	459
Others	Fruits	Vegetables	100	67	0.7	6	0.20	21	6
	100% juice	100% vegetable juice	100	17	0.6	0	0.06	0	0
		100% vegetable & fruit juice	100	43	0.5	1	0.05	3	1
		Fruit juice	100	30	0.6	0	0.05	1	1
	Mild and processed milk	Milk	100	67	3.3	42	0.03	155	124
		Cheese	100	67	4.3	57	0.02	208	195
		Yogurt	100	71	1.1	11	0.00	50	37
	Others	Coffee	100	4	0.2	0	0.00	0	0
		English tea	100	1	0.1	0	0.01	0	0
		Green tea	100	2	0.2	0	0.01	0	0
		Other drink	100	51	0.0	0	0.00	0	0

The **a**-value was defined as the revised index of tyrosine or phenylalanine. The calculation of the revised index is explained in detail next.

Detailed explanation of how to calculate the indices based on amino-acid constituent tables

Based on the dietary intake standards for different age categories of Japanese children, recommended amounts were set for two levels: 2 year olds and 3 to 5 year olds [19]. One-year-old and six-year-old children were excluded from this study, because one-year-olds are often in the post-weaning period and do not have established eating habits and the number of six-year-old respondents was too small.

Representative 'menus’ for well-balanced breakfasts including a staple food, main dish, side dish and other items (soup, beverages) were used for calculating tyrosine and phenylalanine indices. The tyrosine and phenylalanine indices were adjusted based on the frequency of taking the well-balanced breakfast, with staple food (carbohydrates), main dish (protein) and side dish (vitamins and minerals). This adjusted value was calculated by reducing the value from 100% when the well-balanced breakfast was taken every day, to 75% when it was taken 4 or 5 times per week, 50% when taken 2 or 3 times, and into 25% when not taken 0 or only taken once per week.

#### *Questions on mental health*

The integrated and fundamental questionnaire on chronotype, sleep habits and mental health included the following two questions on mental health (Mental Health Scale, [4,13,15]) (Table 4).

**Table 4:** Questions on mental health

**Questions**	**Answers on a four-point scale**			
	**4**	**3**	**2**	**1**
1. How frequently is your child angry due to a small trigger?	Often	Sometimes	Rarely	Never
2. How frequently is your child becoming 'less vigorous’?	Often	Sometimes	Rarely	Never

Scores for 'mental health’ were calculated by adding the numbers of the answers, and ranged from 2 to 8.

#### *Statistical analysis*

The questionnaire data were analyzed with SPSS 12.0 statistical software. The ANCOVA (analysis of covariance) was used for statistical analysis on the relationship between tyrosine and phenylalanine intake and the scores of the Torsvall-Åkerstedt Diurnal Type Scale (Table 1) and mental health scores with covariance of tryptophan intake. In the case of ANCOVA, the diurnal type scale scores and mental health scores were analyzed as continuous variables, and the values for intake of tyrosine, phenylalanine and tryptophan were categorized to less than 800 mg and more than 800 mg, because 13.5% of all infants were taking extreme low amounts(less than 800 mg),which can be called the 'extremely low intake group’. Earlier 24h tracer studies showed that between 30 and 40 mg/kg/day of phenylalanine were required for human consumption [20]. For Japanese infants aged 5 years [21], the required phenylalanine amount was calculated as 18 kg × 40 mg = 720 mg per day, which is similar to the cut-off value of 800 mg in this study. This cut-off value serves as a kind of security, in case the infants could only get one well-balanced meal with staple food (carbohydrates), main dish (protein) and side dish (vitamins and minerals) because of some severe economic condition or educational difficulty (for example neglect of childcare by mothers).

#### *Ethical treatment*

This study followed the ethical guideline of the *Journal of Physiological Anthropology* for conducting research on human subjects. All the parents of infants aged 2 to 5 years who participated gave their consent. The kindergarten teachers’ committees of all kindergartens carried out an ethical inspection of the contents of the questionnaire and gave permission for the administration of this epidemiological study to infants.

## Results

**Figure 2:**
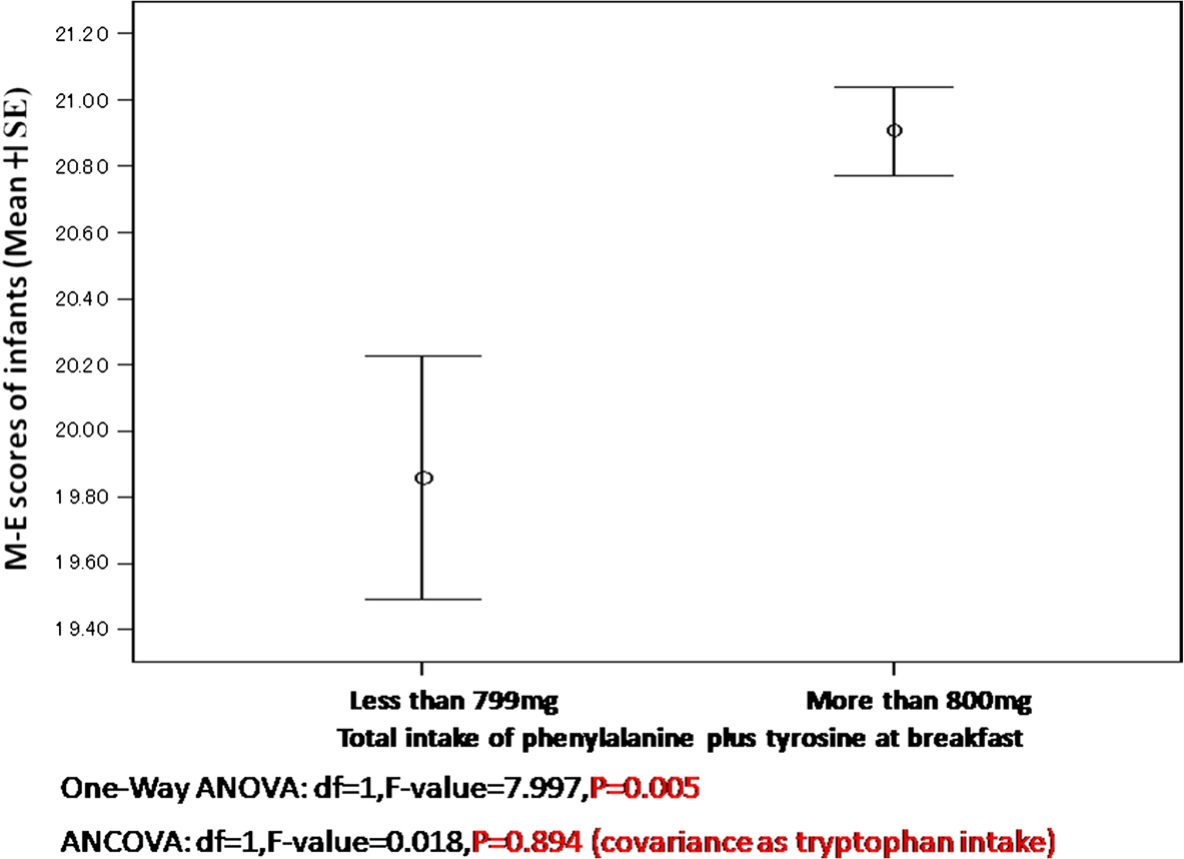
**Intake of phenylalanine plus tyrosine and morningness-eveningness scores in young Japanese children aged 2 to 5 years.** 106 infants: 1 to 799 mg; 669 infants: 800 mg or more. M-E, morningness-eveningness; SE, standard error.

**Figure 3:**
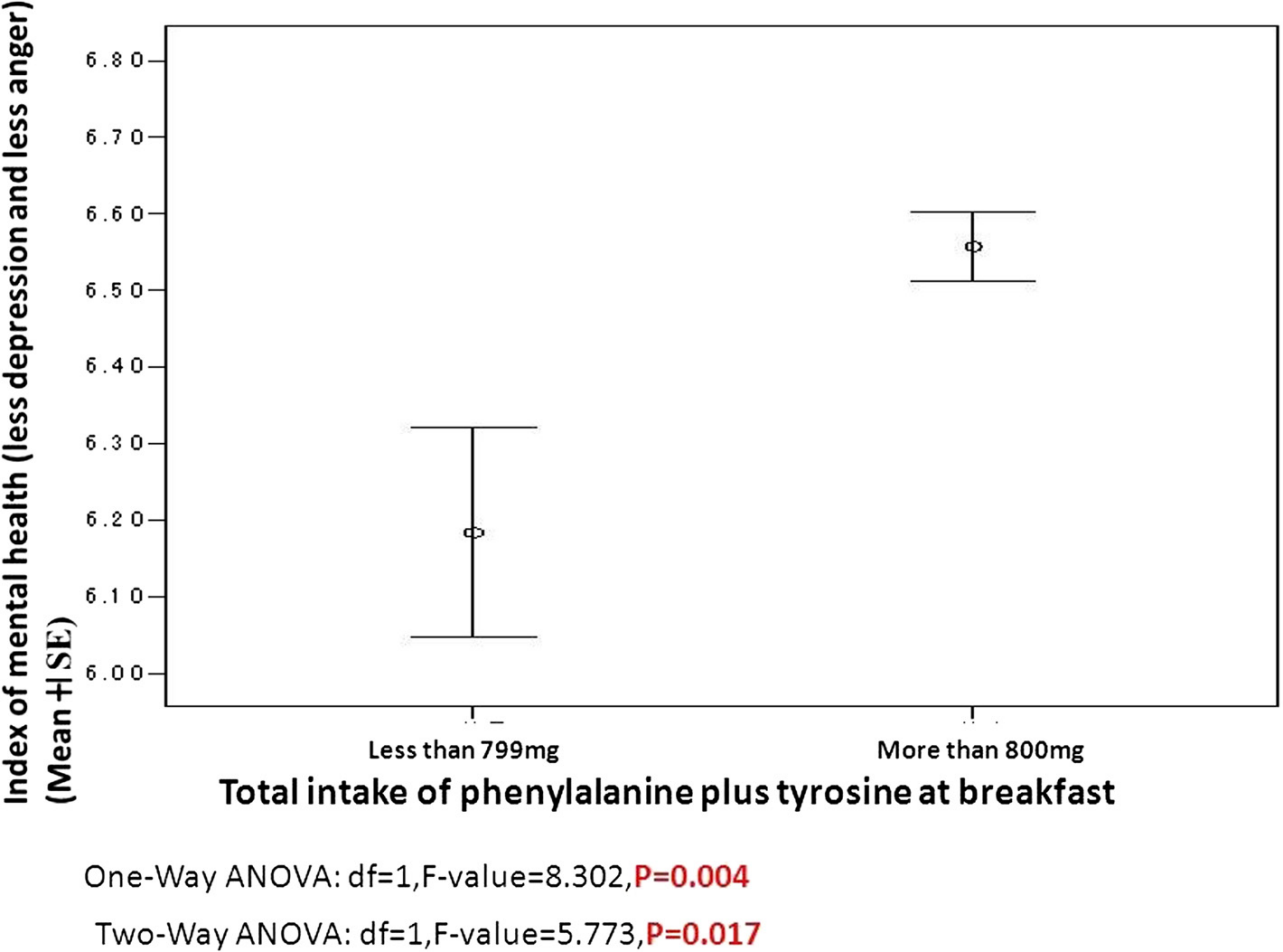
**Intake of phenylalanine plus tyrosine and index of mental health (depression and anger) in Japanese infants.** (106 infants: 1-799 mg; 669 ones: 800 mg or more).

Infants who ingested more than 800 mg (per meal) of tyrosine or phenylalanine at breakfast were more morning-type than those who ingested less than 800 mg (Figure 1). However, this relationship disappeared in the ANCOVA analysis (Figure 2, Table 5). Infants who ingested more than 800 mg of the two amino acids at breakfast showed significantly higher scores of mental health (lower frequency of depression and anger), and this relationship remained significant when ANCOVA was performed with the covariance of the tryptophan amount (Figure 3, Table 5). The average number of days (per week) when the infants took a well-balanced breakfast was 5.19.

**Table 5:** Statistical analysison relationship between catecholamine intake at breakfast, circadian typology, and mental health

	**Morningness-eveningness scores**			**Scores of mental health scale**		
	**d*f***	***F*value**	***P*value**	**d*f***	***F*value**	***P*value**
One-Way ANOVA	1	7.997	0.005	1	8.302	0.004
ANCOVA	1	0.018	0.894	1	5.773	0.017

One-Way ANOVA, variance: catecholamine amount; ANCOVA, variance: catecholamine amount, covariance: tryptophan amount. The ANCOVA results show that the catecholamine relates with mental health but chronotype is related not with the catecholamine but with tryptophan taken at breakfast, from an epidemiological point of view.

## Discussion

Protein includes several functional amino acids, such as aromatic amino acids (tryptophan, tyrosine, and phenylalanine), which relate to brain function, and branched-chain amino acids (valine, leucine, isoleucine) which occupy 30 to 40% of all amino acids making-up skeletal muscles. Infants who took more than 800 mg of phenylalanine or tyrosine were shown (Figure 1) to be more morning-type than those who took less than 800 mg. This significant relationship completely disappeared on ANCOVA analysis with covariance of tryptophan. Therefore, consumption of phenylalanine and tyrosine at breakfast appears to have no relationship with circadian typology, whereas the significance of tryptophan intake via serotonin synthesis might be powerful in Japanese infants. This result supports previous studies on the relationship between tryptophan consumption at breakfast and circadian typology [4,13]. Serotonin in cerebrospinal fluid, especially in the morning, is hypothesized to be a zeitgeber of the biological clock that drives the sleep-wake cycle in human beings.

Intake of phenylalanine and tyrosine in breakfast appears to relate with mental health in Japanese infants aged 2 to 5 years. As this relationship remained even after ANCOVA with covariance of tryptophan, higher mental health might be achieved partially via synthesis of catecholamine, such as dopamine, independently of serotonin synthesis. The results of this study suggest that phenylalanine and tyrosine consumed together at breakfast might be used in catecholamine synthesis in infants. For such aromatic amino acids to function in the central nervous system, they should pass competitively through the blood–brain barrier [22].

In people with depression, the noradrenergic and dopaminergic systems are targets for antidepressants [23]. Changes in catecholaminergic neurotransmission correlated with antidepressant response [24,25], which showed positive neurobiological results under combined treatment with sleep deprivation [26]. For healthy young children, consuming a protein-rich breakfast may be an effective way to prevent depression via the dopaminergic system. In addition, the ratios of plasma tryptophan to large neutral amino acids would be related to the mood modulation in relation to the transporting of amino acids at the blood–brain barrier [27].

Infants taking a breakfast rich in tyrosine and phenylalanine showed strict morning-type lifestyle habits and small differences between weekdays and weekends in wake-up time and bedtime. They might be brought up with strict discipline by parents, although there are no data on discipline. Takeuchi *et al*. [28] showed that Japanese junior-high school students who had had bedtime disciplines in their young childhood were more morning-type than those who had not. Disciplines on infants might be an important factor in affecting their chronotype in the future.

Energy intake in breakfast could be hypothesized to affect mood, mental power, and emotion. However, owing to similar epidemiological data on Japanese young children in 2010, there were no significant relationship between energy intake at breakfast and frequencies of anger and depression (Kruskal-Wallis tests, all *P*> 0.05) (Harada *et al*., unpublished work). At least for young children, calorie intake at breakfast might hardly affect mental health.

This study suggests the possibility that meal composition could be related to circadian typology and mood. However, the reverse could also be possible: evening or depressive people like to get up later and have a bad appetite in the morning, so they tend to have insufficient meals at breakfast. This produces a kind of negative spiral phenomenon.

Further studies are needed to examine consumption of phenylalanine and tyrosine at lunch and dinner, in order to clarify the potential relationship of catecholamine synthesis to circadian rhythms. Young *et al*. [29] used positron emission tomography scans to study 5-HT (serotonin) synthesis in the human brain. This technique can also be used to measure serotonin synthesis in students. It may be possible to examine catecholamine concentration in the cerebrospinal fluid in young and adult experimental animals (rodents and monkeys) in the future.

However, to reach a solid conclusion on this topic, more detailed approach is needed. For example, the experiment to examine whether the deletion and adding of tyrosine and phenylalanine from and to breakfast affects mental health in human beings would be a powerful approach for a solid conclusion. Moreover, the measurement of actual and real amounts of tyrosine and phenylalanine ingested from breakfast meals and their analysis on the relationship between the actual amounts and mental health remain to be done.

## Abbreviations

AMPT: α-methylparatyrosine; ANCOVA: Analysis of covariance; ANOVA: Analysis of variance.

## Competing interests

The authors declare that they have no competing interests.

## Authors’ contributions

OA analyzed data, and drafted this manuscript.KW analyzed data and participated in discussion on the results. TN participated in discussion on the results. NT collected the data and participated in discussion on the results. MK participated in discussion on the results. MN made Tyrosine Index and Phenylalanine Index, analyzed data. HT made Tyrosine Index and Phenylalanine Index, analyzed data. TH supervised this study and drafted this manuscript. All authors read and approved the final manuscript.
